# The Role of Paraoxonase-1 Activity, Apolipoprotein B Levels, and Apolipoprotein B/Apolipoprotein A-I Ratio as Risk Markers for Aortic Stenosis in Patients with a Bicuspid Aortic Valve

**DOI:** 10.3390/antiox14020167

**Published:** 2025-01-30

**Authors:** Maria Kwiatkowska, Agnieszka Mickiewicz, Aleksandra Krzesińska, Agnieszka Kuchta, Maciej Jankowski, Marcin Gruchała, Marcin Fijałkowski

**Affiliations:** 1 1st Department of Cardiology, Medical University of Gdańsk, 80-211 Gdańsk, Poland; marianowak@gumed.edu.pl (M.K.); mgruch@gumed.edu.pl (M.G.); marcin.fijalkowski@gumed.edu.pl (M.F.); 2 Department of Clinical Chemistry, Medical University of Gdańsk, 80-211 Gdańsk, Poland; aleksandra.krzesinska@gumed.edu.pl (A.K.); agnieszka.kuchta@gumed.edu.pl (A.K.); majank@gumed.edu.pl (M.J.)

**Keywords:** bicuspid aortic valve, aortic stenosis, apolipoprotein B, apolipoprotein A-I, paraoxonase-1

## Abstract

The bicuspid aortic valve (BAV) is commonly associated with the early degeneration of the aortic valve. Up to 45% of BAV patients over the age of 50 develop aortic stenosis (AS). Although published data indicate a robust interplay between lipids and calcific AS in tricuspid aortic valve patients, the studies on the BAV population are lacking. We aimed to evaluate the association between selected lipid markers and the occurrence of AS in BAV patients. Methods: The study included 76 adults (21 female) with a BAV diagnosed by echocardiography, divided by age and AS diagnosis. Biochemical parameters concentrations in serum were measured: high density lipoprotein cholesterol (HDL-C) levels by standard enzymatic colorimetric tests, low density lipoprotein cholesterol (LDL-C) levels by the Friedewald formula, apolipoprotein A-I (Apo AI) and apolipoprotein B (Apo B) serum concentration by the nephelometric method, and paraoxonase-1 activity (PON-1 ASE) and arylesterase activity (PON-1 ARE) based on paraoxon and phenyl acetate hydrolysis. Results: A total of 54 patients (15 female) were more than 45 years old and 22 (6 female) were 45 or less years old. BAV patients with AS aged ≤45 had higher levels of Apo B, compared to those without AS [110.5 (102–132) vs. 95.6 (77–101) mg/d; *p* 0.044]. Similarly, Apo B/Apo AI ratio was higher in BAV patients with AS aged ≤45, compared to those without AS [(0.8 (0.7–1) vs. 0.6 (0.5–0.7); *p* 0.029]. In the group aged ≤45, Apo B showed a positive correlation with the aortic valve peak transvalvular velocity (AV Vmax) measurement (R Spearman 0.6, *p* 0.004). We found also that, among young BAV patients, those with AS had a lower level of PON-1 ARE compared to the cohort without AS [63.4 (52–80) vs. 85.3 (70–102); *p* 0.012]. We did not find any differences in lipid parameters in patients aged >45. Conclusions The metabolic link between Apo B level and Apo B/AI ratio with AS presence in BAV patients under 45 years of age suggests a significant impact of these parameters on the earlier development of AS in the BAV population. Molecules associated with high density lipoprotein and its antioxidant function, such as PON1, are valuable markers for AS development, compared to HDL-C and LDL-C levels.

## 1. Introduction

Bicuspid aortic valve (BAV) is a congenital valvular defect that affects approximately 0.5–2% of the general adult population [1]. The abnormal structure of the leaflets leads to the development of aortic valve dysfunction, both in the form of regurgitation and stenosis. Furthermore, an aortic aneurysm often accompanies this condition [2]. As a consequence, patients with a BAV frequently and at a young age require qualification for cardiac surgery. In the registry from Olmsted County in Minnesota, observing over 200 individuals with a BAV, the mean age at the time of cardiac surgery was 40 ± 20 years compared to 67 ± 16 years for patients with a tricuspid aortic valve [3,4]. Overall, up to 50 percent of BAV individuals undergo aortic valve replacement (AVR) [5]. Aortic stenosis (AS) is a frequent cause of surgery in this group. Calcification of the cusps often occurs by the age of 40 [4]. The BAV is associated with the occurrence of degenerative AS at a significantly younger age compared to the population with a tricuspid aortic valve [5,6].

On the other hand, the BAV population remains a heterogenous group, and the occurrence and course of valve defects in these patients remain unpredictable. While a BAV may lead to advanced calcification of the aortic valve, we do not observe this process in all BAV individuals. Therefore, among the BAV population, factors that may be predictors for the calcified AS have been investigated. Studies evaluating the association between the morphological type of BAV and the occurrence of valve stenosis have not yielded consistent results [6,7]. In a meta-analysis involving over 4000 patients, Zhenzhen Mai indicated that a type of BAV with right-coronary and non-coronary cusps fusion most frequent predisposes to AS [8]. However, earlier registries did not demonstrate similar results [9]. Uncertainties in the relationship between aortic valve cusps fusion morphology and the development of AS highlight the need to focus on modifiable risk factors for valve calcification.

The impact of lipid metabolism disorders on the progression of calcified AS has been demonstrated in tricuspid aortic valve populations [10,11]. High levels of lipids, especially low density lipoprotein cholesterol (LDL-C), stimulate a chronic inflammation of the endothelium resulting in progressive fibrosis and further reduction in the aortic valve area. It has been shown that apolipoprotein B (Apo B), the main and only essential structural protein component of low density lipoprotein (LDL), is a more precise marker for assessing cardiovascular risk than LDL-C levels. Plasma concentration of apo B is a reliable indicator of the number of atherogenic particles that contribute to the development of atherosclerosis. Serum Apo B concentrations are also associated with the AS development [12].

The role of high density lipoprotein cholesterol (HDL-C) levels in preventing atherosclerosis and endothelial dysfunction has been investigated [13]. Recent studies conducted in patients with tricuspid aortic valve suggest that HDL-C may also play a significant protective role in the pathogenesis of AS [14]. This connection underscores broader cardiovascular benefits of high density lipoprotein (HDL) beyond its traditional anti-atherosclerotic mechanisms. HDL has a multifaceted range of protective functions, including neutralizing free radicals and oxidized lipoproteins, facilitating reverse cholesterol transport, and demonstrating potent anti-inflammatory properties [15]. These mechanisms not only inhibit vascular damage but also halt the processes of degeneration and calcification in the aortic valve [16]. Traditionally, serum HDL-C levels have served as a readily accessible biomarker for cardiovascular risk assessment. However, elevated HDL-C levels do not always correlate with its protective function [17]. Moreover, high HDL-C levels may paradoxically exhibit a pro-inflammatory effect [18]. Increasingly, the evaluation of HDL antioxidant properties is recognized as a more precise indicator of cardiovascular risk.

A biomarker associated with HDL, paraoxonase-1 (PON-1), an enzyme exhibiting antioxidant properties and reducing the oxidation of LDL-C, have been studied among patients with AS. Several different activities of PON-1 were described with research mainly focusing on PON-1 paraoxonase (PON-1 ASE) and arylesterase activity (PON-1 ARE), and their role in the development of cardiovascular disease [19] Z. Wang has demonstrated, on over hundred patients who underwent AVR, that a lower PON-1 level in aortic valve tissue may be associated with the progression of calcification in AS [20].

Another marker linked to HDL is apolipoprotein A-I (Apo AI), the main protein component of HDL. Apo AI plays a key role in cholesterol’s transport, facilitates the efflux of cholesterol from macrophages in the arterial endothelium, supports antioxidant processes and regulation of inflammatory reactions, and has been shown an important indicator of the risk of AS occurrence [21,22].

The Apo B/Apo AI ratio represents the proportion between atherogenic and antiatherogenic lipoproteins in plasma. Thus, it constitutes an accurate marker of cardiovascular risk [23]. A correlation between this ratio and the occurrence of AS was also demonstrated. In a study involving the EPIC-Norfolk cohort, Kang H Zheng’s et al. demonstrated a correlation between the Apo B/Apo AI ratio and the incidence of AS [24].

Patients with a bicuspid and tricuspid aortic valve may differ in terms of their lipid parameters [25,26]. Moreover, BAV patients may be at higher risk of atherosclerosis and coronary artery disease occurrence [27]. Finally, BAV patients are not a homogenous group. The studies profiling the BAV population based on aortic valve function and lipid markers are lacking.

Therefore, we aimed to assess the relationship between the serum concentration of selected lipid markers and the occurrence of AS among BAV patients in different age groups.

## 2. Material and Methods

### 2.1. Group Characteritics

Adult patients with a BAV, diagnosed by echocardiography and admitted to the First Clinic of Cardiology of Medical University of Gdansk, were enrolled into the study. Exclusion criteria included active inflammatory process, autoimmune diseases, active oncological disease, hyperthyroidism, pregnancy, unstable clinical condition (clinical features of circulatory or respiratory failure), and familial hypercholesterolemia. Patients were divided into two age groups with an age burden of 45 years. Due to the assessment of the atherogenic markers impact on the occurrence of AS, this division into age groups was established based on the age burden defining young coronary artery disease [28,29]. Subsequently, each age group was divided into two subgroups depending on AS occurrence. Clinical data including age, anthropometric data (weight and height), chronic diseases (such as hypertension, diabetes mellitus, coronary artery disease), smoking habit, and statin therapy were collected during the medical interview. The study protocol was approved by the Local Bioethics Committee at the Medical University of Gdansk (protocol no. NKBBN/487-517/2019). Written informed consent was obtained from each patient prior to their inclusion in the study.

### 2.2. Echocardiographic Examination

Transthoracic examination was performed in each case and included following parameters: left ventricle ejection fraction (LVEF), aortic valve area (AVA), aortic valve peak transvalvular velocity (AV Vmax), mean transvalvular pressure gradient (PGmean); aortic regurgitation evaluation, aortic complex diameters. AS and aortic regurgitation were defined according to European Society of Cardiology Guidelines from 2021 [30]. Aortopathy was defined by an aortic root or ascending aorta diameter more than 40 mm. Patients with at least a mild stage of AS were included in the AS subgroups.

### 2.3. Biochemical Analysis

Peripheral blood samples were drawn from each patient between 7 and 8 a.m. following the overnight fast. The serum was separated by centrifugation at 1000× *g* for 15 min and was stored at −80 °C pending analysis. Total cholesterol (TC), HDL-C, and triglycerides (TG) were measured in serum using standard enzymatic colorimetric tests (Wiener Lab, Warsaw, Poland). LDL-C was calculated using the Friedewald formula (there were no cases of TG elevation above 400 mg/dL) [31,32]. The apolipoprotein (Apo AI and Apo B) serum concentrations were determined using the nephelometric method with antibodies obtained from Siemens Healthcare Diagnostics (Eschborn, Germany) on a Behring laser nephelometer. The PON-1 ASE and PON-1 ARE activities were analyzed in serum based on paraoxon and phenyl acetate hydrolysis, respectively, according to the procedure described earlier [33].

### 2.4. Statistic Analysis

All data were collected and computed in MS Excel and a standard statistical package. Variables with normal distribution were compared with the Student’s *t*-test; non-normally distributed data were compared with the U Mann–Whitney test. Dichotomous variables were compared with Chi-squared test with Yates’s correction for continuity. Correlation for data with non-normally distribution was calculated by the Spearman correlation. For selected biomarkers, the area under the curve (AUC) was calculated using receiver operating characteristic (ROC) analysis, and the optimal cut-off values corresponding to the minimal false positive and false negative parameters were determined. The continuous variables were expressed as medians with 25th and 75th percentiles. *p*-value less than 0.05 was considered valid for all tests.

## 3. Results

A total of 76 patients (21 female) were enrolled into the study. Clinical characteristics of the studied group are presented in Table 1 and Table 2. The average age of observed patients was 53.7 ± 13 years. A total of 54 patients (15 female) were in the age category of more than 45 years. A total of 22 patients (6 female) were aged 45 or younger. In the group aged >45, there were 35 patients (65%) with AS, and in the group aged ≤45, there were 9 patients (41%) with AS. The group aged >45 had significantly more frequent arterial hypertension (67% vs. 14% of patients; *p* < 0.001), coronary artery disease (35% vs. 5% of patients; *p* 0.014), and frequently required statins therapy (50% vs. 18% of patients; *p* 0.021) compared to patients aged ≤45 (Table 1).

In the group aged ≤45, there were no significant differences in terms of sex, age, body mass index, statin use, and the prevalence of hypertension, smoking, coronary artery disease, or diabetes mellitus depending on the presence of AS. On the contrary, in the cohort aged >45, patients with AS were characterized by their older age and more frequently requiring statins compared to patients without AS (Table 2).

### 3.1. Echocardiographic Characteristics of Groups

The prevalence of AS and aortopathy was similar in both age groups. Moderate to severe aortic regurgitation was more frequently observed in patients aged ≤45. Function of the left ventricle calculated by LVEF was in a similar range for both age groups (Table 3).

In the group aged >45, patients with AS and without AS differed in the frequency of aortic regurgitation occurrence. In the group aged ≤45, there was no significant difference in terms of LVEF, aortic regurgitation, and aortopathy prevalence depending on AS diagnosis (Table 4).

Among patients with AS, older patients aged >45 had lower AVA compared to those aged ≤45 [AVA 0.9 (0.8–1.2) cm^2^ vs. 1.3 (0.9–1.4) cm^2^; *p* 0.023]. We found no differences in AV Vmax and PGmean measurements (Table 5).

### 3.2. Biochemical Analysis

**Figure 1:**
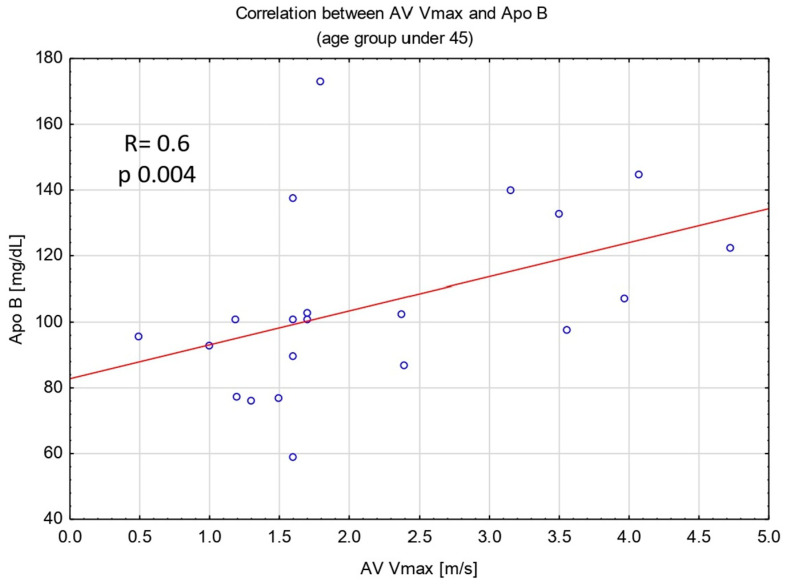
Spearman’s rank correlation between AV max measurement and Apo B level. Abbreviations: AV Vmax—aortic valve peak transvalvular velocity, Apo B—apolipoprotein B.

**Figure 2:**
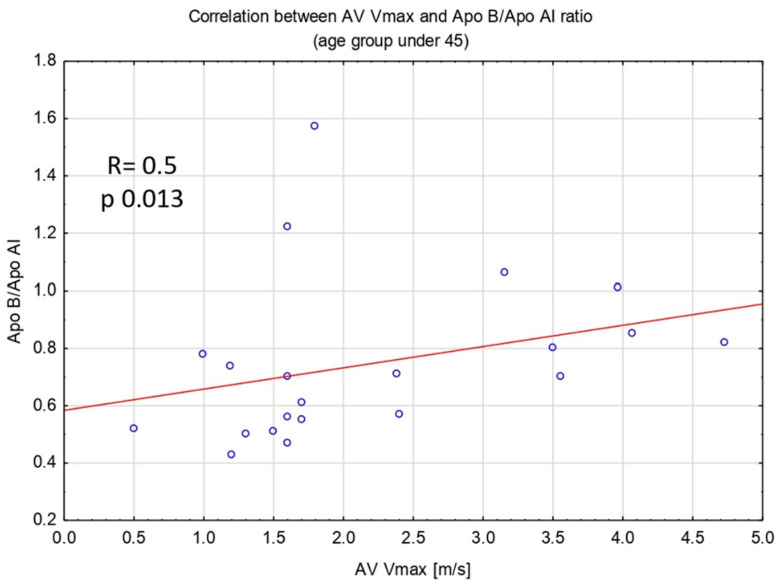
Spearman’s rank correlation between AV max measurement and Apo B/Apo AI ratio. Abbreviations: AV Vmax—aortic valve peak transvalvular velocity, Apo B/Apo AI—apolipoprotein B/apolipoprotein A-I ratio.

Patients aged ≤45 with AS in comparison to those without AS had significantly higher levels of Apo B [110.5 (102–132) vs. 95.6 (77–101) mg/dL; *p* 0.044] and Apo B/Apo AI ratio [(0.8 (0.7–1) vs. 0.6 (0.5–0.7); *p* 0.029]. We found no differences in the level of TC, HDL-C, LDL-C, and TG. Moreover, in the group aged ≤45, Apo B level and Apo B/Apo AI ratio showed a positive correlation with AV Vmax measurement (R Spearman was 0.6 for Apo B and AV Vmax, and 0.5 for Apo B/Apo AI and AV Vmax, *p* < 0.05 for both analyses) (Figure 1 and Figure 2). Furthermore, in the group aged ≤45, patients with AS had significantly lower activity of PON-1 ARE compared to those without AS [63.4 (52–80) vs. 85.3 (70–102) U/L; *p* 0.0124].

**Figure 3:**
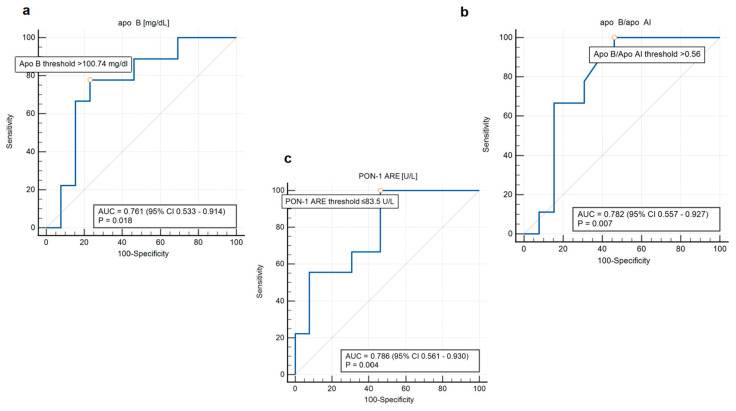
ROC curves of Apo B levels (**a**), Apo B/Apo AI ratio (**b**), PON-1 ARE (**c**) activity and the presence of AS for patients aged ≤45. Abbreviations: AUC—Area Under the Curve; CI—Confidence Interval; Apo B—apolipoprotein B; Apo AI—apolipoprotein AI; PON-1 ARE—arylesterase activity.

Among patients aged ≤45, the analysis for Apo B levels, Apo B/Apo AI ratio, and PON-1 ARE activity was extended to ROC analysis. AUC for Apo B was 0.761 [95% confidence interval (CI): 0.533 to 0.914; *p* 0.018], for Apo B/Apo AI ratio 0.782 (95% CI: 0.557 to 0.927; *p* 0.007), indicating a moderate to good ability to discriminate between patients with and without AS. PON-1 ARE activity demonstrated the highest AUC of 0.786 (95% CI: 0.561 to 0.930; *p* 0.004), indicating the best discriminatory power among the biomarkers tested (Figure 3).

Among patients aged >45, there were no differences in analyzed parameters depending on AS prevalence.

All results from the biochemical analysis were presented in Table 6.

## 4. Discussion

The findings of our study showed an association between AS and molecules associated with lipid metabolism, such as Apo B, Apo B/Apo AI ratio, and PON1-ARE in BAV patients at age ≤45. Moreover, the Apo B level and Apo B/Apo AI ratio correlated with the echocardiographic parameter of AS severity such as AV Vmax.

Our results suggest that lipid metabolism may play a significant role in the early development of calcific AS among patients with a BAV, which is a frequent BAV complication, affecting up to 45% of patients over the age of 50 [34]. It is also the most common cause of qualifying for aortic valve replacement in the BAV adult population [35]. Furthermore, patients with a BAV required surgery due to AS at a significantly younger age than patients with a tricuspid aortic valve [36]. In the study of a large Australian cohort by Michelle S Lim’s team, the mean age of BAV patients referred to AVR due to AS was 55 ± 17 years (22 years earlier than patients with tricuspid aortic valve) [37]. The exact origin of early calcification and accelerated development of AS in patients with BAV remains unclear. One possible hypothesis considers mechanical factors, such as endothelial damage of valve cusps caused by abnormal blood flow dynamics [38]. However, this anatomical aspect does not fully explain why only a subset of the BAV population experience progression to significant valve calcification, while some individuals remain without a valvular dysfunction throughout their life.

Consequently, attention has also been directed towards a risk factor for calcific AS that overlaps with those for atherosclerosis, including hyperlipidemia, hypertension, and diabetes mellitus [39]. Studies suggest that patients with a BAV constitute a heterogeneous group regarding their lipid profiles. For instance, Elena Sticch et al. showed that an elevated level of lipoprotein (a) in patients with BAV was associated with the degree of aortic valve calcification [40]. Additionally, it has been shown that LDL-C and Apo B levels may significantly affect the progression of BAV in case of aortopathy. [41] These findings underline the need for further investigation into the role of specific lipid markers in the early manifestation of BAV complications.

The second observation derived from our results highlights the need for a broader analysis of lipid markers. To date, the relationship between LDL-C levels and the risk of both atherosclerosis and AS has been well demonstrated [42,43]. The beneficial effect of high HDL-C on the occurrence of calcific AS or cardiac events is less clear [44,45]. Nevertheless, HDL-C and LDL-C levels are the most commonly used markers of cardiovascular risk assessment, though they do not always adequately reflect lipid balance [18,46].

Apo B is a protein found in all atherogenic lipoproteins. Studies have shown that Apo B levels may be a more precise marker of cardiovascular risk than LDL-C [47]. It should be noted that the measurement of Apo B is less dependent on triglyceride (TG) levels and fasting status, making it a more stable parameter than LDL-C [48]. The assessment of HDL properties, including its associated molecules, appears to better explain its role in both the development of atherosclerosis and AS. The main protein component of HDL is Apo AI [49]. The Apo B/Apo AI ratio enables the assessment of the balance between atherogenic and protective lipoproteins, making it an important marker for evaluating cardiovascular risk [50]. Ivert et al. proved an association between elevated Apo B/Apo AI ratio and increased appearance of AS among patients with tricuspid aortic valve [10]. Our findings demonstrate a similar observation among young individuals with BAV. Patients aged ≤45 with AS had increased concentrations of Apo B and Apo B/Apo AI ratio. We did not observe any differences in the levels of LDL-C and HDL-C. In our study, the cut-off value for Apo B obtained from the ROC analysis was 100.74 mg/dl, a value that corresponds to an increased cardiovascular risk [51]. The cut-off value for Apo B/Apo AI ratio was 0.56. For comparison, in the AMORIS and INTERHEART studies, the Apo B/Apo AI ratio below 0.7 for men and 0.6 for women was associated with low cardiovascular risk. Nevertheless, it should be noted that there are no clearly established cut-off values for Apo B levels and the Apo B/Apo AI ratio in the context of AS, and further research is needed in this area.

Another marker associated with HDL is PON-1, which demonstrates antioxidant properties. Although the relationship of its different activities and the occurrence of AS is less well understood, some studies have shown a correlation between low activity of PON-1 and the severity of AS [52]. In our study, patients with a BAV and AS at ≤45 years of age showed significantly lower activity of PON-1 ARE compared to those without AS, with the cut-off value below 83.5 U/L. The difference was observed despite the fact that the level of Apo AI did not differ between these groups. This relationship underscores the potential significance of the marker in various manifestations of BAV complications and emphasizes the need for further research to determine PON-1 activity values associated with the risk of AS development. By analyzing the antioxidant components of HDL and its associated biomarkers, we can gain a deeper understanding of HDL quality and functionality, which may provide a more accurate assessment of its cardiovascular protective capacity [17,53].

Interestingly, we did not observe similar relationships among patients above 45 years of age. However, it should be noted that in this group, the etiology of AS may be more multifactorial, considering the influence of age. Additionally, these patients more frequently had diagnosed hypertension and chronic coronary syndrome. It should be noted that in the group aged >45, patients with AS more often required statin treatment, which could have significantly affected the obtained results.

The main limitation of the current study is relatively small sample size, drawn from a single-center population, which may have reduced the statistical power of the analysis. Additionally, a longer follow-up period is required to assess the impact of Apo B levels, Apo B/Apo A1 ratio, and PON1 activity on the progression of AS in the BAV population. The study did not consider dietary habits or the dosage of lipid-lowering medications, which also may have affected the evaluated markers. However, the cohort aged ≤45 was homogeneous in terms of age, sex, and the chronic conditions included in the study; the older group exhibited greater heterogeneity. Patients aged >45 with AS were significantly older and more frequently used statins compared to those without AS. However, we would like to highlight that, to the best of our knowledge, this is the first prospective study in BAV patients which indicated that Apo B levels, Apo B/Apo A1 ratio, and PON-1 activity are associated with AS appearance. Our results require further prospective studies in larger cohorts.

## 5. Conclusions

In summary, the AS occurrence among the BAV population has complex etiology and is challenging to predict. Our results suggest a potential metabolic link between Apo B level and Apo B/Apo AI ratio and the occurrence of AS in patients with a BAV. Notably, this association was observed only in patients up to 45 years of age. Furthermore, our findings underscore the value of investigating molecules associated with HDL and its antioxidant function, such as PON1, as a useful marker for AS development, compared to traditionally measured HDL-C and LDL-C levels.

## Figures and Tables

**Table 1:** Clinical characteristics of BAV patients age ≤ 45 and age > 45.

	Age ≤ 45	Age > 45	*p* Value
No (female)	22 (6)	54(15)	0.812 *
Age	39.5 (33–42)	60.5 (52–68)	<0.001 **
BMI [kg/m^2^]	26.6 (23–29)	27.8 (25–31)	0.145 **
Smoking	5 (23%)	19 (35%)	0.431 *
Hypertension	3 (14%)	36 (67%)	<0.001 *
Diabetes mellitus	1 (5%)	8 (15%)	0.387 *
Coronary artery disease	1 (5%)	19 (35%)	0.014 *
Statin therapy	4 (18%)	27 (50%)	0.021 *

Abbreviations: BMI—body mass index. * Chi-squared test with Yates’s correction, ** Mann–Whitney U test.

**Table 2:** Comparison of clinical characteristics of patients in two age groups based on the AS presence.

	Age ≤ 45			Age > 45		
	No AS	AS	*p* Value	No AS	AS	*p* Value
No (female)	13 (2)	9 (4)	0.309 *	19 (3)	35 (12)	0.258 *
Age	39 (31–41)	40 (34–43)	0.378 ***	55 (49–61)	65 (59–69)	0.002 **
BMI [kg/m^2^]	27 (24–32)	25 (23–27)	0.190 ***	27 (25–30)	28 (26–31)	0.340 ***
Smoking	3 (23%)	2 (22%)	0.638 *	7 (37%)	12 (34%)	0.912 *
Hypertension	2 (15%)	1 (11%)	0.730 *	13 (68%)	23 (66%)	0.920 *
Diabetes mellitus	1(8%)	0	0.845 *	2 (10%)	6 (17%)	0.801 *
Coronary artery disease	0	1(11%)	0.845 *	6 (32%)	13 (37%)	0.912 *
Statin therapy	3 (2%)	1(11%)	0.878 *	5 (26%)	22 (63%)	0.023 *

Abbreviations: BMI—body mass index. * Chi-squared test with Yates’s correction, ** Mann–Whitney U test, *** Student’s *t*-test.

**Table 3:** Echocardiographic assessment of BAV complications and LVEF according to age.

	Age ≤ 45	Age > 45	*p* Value
LVEF [%]	60 (35–65)	60 (55–64)	0.951 **
AS	9 (41%)	35 (65%)	0.097 *
AR	13 (59%)	14 (26%)	0.013 *
Aortopathy	13 (59%)	37 (69%)	0.604 *

Abbreviations: LVEF—left ventricular ejection fraction, AS—aortic stenosis, AR—moderate to severe aortic regurgitation. * Chi-squared test with Yates’s correction, ** Mann–Whitney U test.

**Table 4:** Echocardiographic assessment of BAV complications and LVEF in two age groups based on the AS presence.

	Age ≤ 45			Age > 45		
	No AS	AS	*p* Value	No AS	AS	*p* Value
LEVF [%]	60 (40–64)	60 (32–70)	1.000 **	60 (55–64)	60 (51–64)	0.926 **
AR	7 (54%)	6 (67%)	0.873 *	9 (47%)	5 (14%)	0.020 *
Aortopathy	8 (61%)	5 (56%)	0.873 *	17 (89%)	27 (77%)	0.455 *

Abbreviations: LVEF—left ventricular ejection fraction, AS—aortic stenosis, AR—moderate to severe aortic regurgitation. * Chi-squared test with Yates’s correction, ** Mann–Whitney U test.

**Table 5:** Echocardiographic characteristics of patients with aortic stenosis according to age.

	Age ≤ 45 + AS	Age > 45 + AS	*p* Value
AVA	1.3 (0.9–1.4)	0.9 (0.8–1.2)	0.023 **
AV Vmax	3.5 (2.8–4.1)	3.9 (3.4–4.4)	0.161 *
AV PGmean	32 (16–46)	40 (25–46)	0.457 *

Abbreviations: AVA—aortic valve area, AV Vmax—aortic valve peak transvalvular velocity, AV PGmean—mean transvalvular pressure gradient. * Student’s *t*-test, ** Mann–Whitney U test.

**Table 6:** Lipid parameters in BAV patients according to age and AS diagnosis.

	Age ≤45			Age > 45		
	No AS	AS	*p* Value	No AS	AS	*p* Value
Apo AI [mg/dL]	150 (126–168)	144 (131–151)	0.371 *	167 (139–184)	156 (146–173)	0.306 *
Apo B [mg/dL]	95 (77–101)	110 (102–132)	0.044 **	100 (93–114)	83 (77–106)	0.144 *
Apo B/Apo AI	0.6 (0.5–0.7)	0.8 (0.7–1)	0.029 **	0.6 (0.5–0.7)	0.6 (0.5–0.7)	0.096 *
TC [mg/dL]	153 (139–166)	150 (126–164)	0.640 **	144 (124–167)	128 (113–147)	0.065 **
HDL-C [mg/dL]	51 (47–54)	48 (45–52)	0.344 *	51 (47–61)	50 (42–58)	0.492 *
LDL-C [mg/dL]	101 (98–114)	107 (94–111)	0.358 **	99 (91–120)	86 (80–106)	0.056 **
LDL-C/HDL-C	2.1 (1.7–2.3)	2.2 (1.9–2.3)	0.860 *	1.9 (1.7–2.2)	1.8 (1.5–2.2)	0.322 **
non HDL-C/HDL-C	2.1 (1.6–2.4)	2.0 (1.9–2.4)	0.874 *	1.7 (1.4–2.2)	1.6 (1.4–2)	0.259 *
TG [mg/dL]	98 (47–113)	63 (52–108)	0.738 **	105 (69–138)	87 (68–113)	0.269 *
PON-1 ASE [U/L]	70 (55–151)	127 (39–163)	0.894 **	89 (53–154)	59 (47–147)	0.484 **
PON-1 ARE [U/L]	85 (70–102)	63 (52–80)	0.012 *	82 (71–99)	79 (59–88)	0.126 **

Abbreviations: Apo AI—apolipoprotein A-I; Apo B—apolipoprotein B; Apo B/Apo AI—apolipoprotein B/Apolipoprotein A-I ratio; TC—total cholesterol; HDL-C—high density lipoprotein cholesterol; LDL-C—low density lipoprotein cholesterol; non HDL-C—non-HDL cholesterol, TG—triglycerides; PON-1 ASE—paraoxonase activity; PON-1 ARE-arylesterase activity. * Student’s *t*-test, ** Mann–Whitney U test.

## Data Availability

The original contributions presented in this study are included in the article/Supplementary Material. Further inquiries can be directed to the corresponding author.

## Supplementary Materials

The following supporting information can be downloaded at: https://www.mdpi.com/article/10.3390/antiox14020167/s1, Table S1: Receiver operating characteristic (ROC) analysis of lipid markers and the presence of aortic stenosis for patients aged ≤45; Table S2: Receiver operating characteristic (ROC) analysis of lipid markers and the presence of aortic stenosis for patients aged >45.
