# Supplementary material for: The Role of Paraoxonase-1 Activity, Apolipoprotein B Levels, and Apolipoprotein B/Apolipoprotein A-I Ratio as Risk Markers for Aortic Stenosis in Patients with a Bicuspid Aortic Valve
Source: Antioxidants (Basel). 2025 Jan 30;14(2):167. doi: 10.3390/antiox14020167 (PMC11851860; doi:10.3390/antiox14020167)
Supplement: Supplementary file 1 [file antioxidants-14-00167-s001.zip › antioxidants-3398001-supplementary.pdf]

Table S1. Receiver operating characteristic (ROC) analysis of lipid markers and the presence of aortic stenosis for patients aged ≤45.

Patients aged ≤45	p	AUC	95% CI	Youden index J
**Apo AI**	0.4850	0.590	0.362 to 0.792	0.2308
**Apo B**	0.0177	0.761	0.533 to 0.914	0.5470
**Apo B/Apo AI**	0.0070	0.782	0.557 to 0.927	0.5385
**TC**	0.6347	0.564	0.339 to 0.771	0.2222
**HDL-C**	0.5619	0.577	0.351 to 0.782	0.2393
**LDL-C**	0.9754	0.504	0.286 to 0.721	0.2479
**TG**	0.7247	0.547	0.323 to 0.757	0.2051
**PON-1 ASE**	0.8818	0.521	0.301 to 0.736	0.3590
**PON-1 ARE**	0.0042	0.786	0.561 to 0.930	0.5385

Abbreviations: Apo AI - apolipoprotein A-I; Apo B - apolipoprotein B; Apo B/Apo AI - apolipoprotein B/Apolipoprotein A-I ratio; TC - total cholesterol; HDL-C - high-density lipoprotein cholesterol; LDL-C - low-density lipoprotein cholesterol; TG - triglycerides; PON-1 ASE - paraoxonase activity; PON-1 ARE-arylesterase activity; AUC - Area Under the Curve; CI - Confidence Interval.

Table S2. Receiver operating characteristic (ROC) analysis of lipid markers and the presence of aortic stenosis for patients aged >45.

Patients aged >45	p	AUC	95% CI	Youden index J
**Apo AI**	0.6739	0.540	0.393 to 0.682	0.2299
**Apo B**	0.0635	0.650	0.500 to 0.781	0.4136
**Apo B/Apo AI**	0.3618	0.575	0.427 to 0.714	0.2727
**TC**	0.0510	0.655	0.512 to 0.780	0.3142
**HDL-C**	0.4131	0.569	0.424 to 0.705	0.2353
**LDL-C**	0.0527	0.665	0.517 to 0.792	0.4028
**TG**	0.2819	0.595	0.450 to 0.729	0.2353
**PON-1 ASE**	0.4696	0.561	0.415 to 0.700	0.3235
**PON-1 ARE**	0.1106	0.633	0.487 to 0.764	0.2647

Abbreviations: Apo AI - apolipoprotein A-I; Apo B - apolipoprotein B; Apo B/Apo AI - apolipoprotein B/Apolipoprotein A-I ratio; TC - total cholesterol; HDL-C - high-density lipoprotein cholesterol; LDL-C - low-density lipoprotein cholesterol; TG - triglycerides; PON-1 ASE - paraoxonase activity; PON-1 ARE-arylesterase activity; AUC - Area Under the Curve; CI - Confidence Interval.
